# FACl as a Bifunctional Additive to Enhance the Performance of Lead-Free Antimony-Based Perovskite Solar Cells

**DOI:** 10.3390/mi16040379

**Published:** 2025-03-27

**Authors:** Xinyu Gao, Zihao Gao, Zhen Sun, Ping Song, Xiyuan Feng, Zhixin Jin

**Affiliations:** 1 School of Science, Yanshan University, Qinhuangdao 066004, China; 2 School of Microelectronics, Northwestern Polytechnical University, Xi’an 710129, China

**Keywords:** antimony-based perovskite solar cells, buried interface, Cs_3_Sb_2_I_9_

## Abstract

Lead halide perovskite solar cells (PSCs) have shown tremendous progress in the last few years. However, highly toxic Pb and its instability have restricted their further development. On the other hand, antimony-based perovskites such as cesium antimony iodide (Cs_3_Sb_2_I_9_) have shown high stability but low power conversion efficiency (PCE) due to the limited transfer of photocarriers and the poor quality of films. Here, we present a novel method to improve the performance of Cs_3_Sb_2_I_9_ PSCs through a FACl-modified buried interface. FACl acts as a bi-functional additive, and FA incorporation enhances the crystallinity and light absorption of films. Furthermore, treatment with FACl optimizes the level position of Cs_3_Sb_2_I_9_. In addition, transient photovoltage and transient photocurrent were employed to confirm the reduction of charge recombination and superior carrier transportation. By using a planar device structure, we found the PCE of a FACl–Cs_3_Sb_2_I_9_-based device to be 1.66%. The device, stored for 2 months under N_2_ conditions, showed a negligible loss in PCE. Overall, this study provides a new strategy to further enhance the performance of Sb-based PSCs.

## 1. Introduction

Over the past decade, lead (Pb) halide perovskite solar cells (PSCs) have achieved remarkable progress, with power conversion efficiencies (PCEs) now exceeding 26% [1]. Despite the outstanding optoelectronic properties of Pb-based perovskites, the inherent toxicity of Pb poses significant environmental and health concerns, potentially hindering their widespread practical application. Consequently, the development of low-toxicity and environmentally friendly alternatives to Pb has become a critical focus for the next generation of Pb-free PSCs. Among the various Pb-free candidates, tin (Sn)-based perovskites have garnered considerable attention, demonstrating PCEs of over 14%. However, the susceptibility of Sn^2+^ to oxidation in ambient conditions presents a major challenge to their stability. In contrast, bismuth (Bi) and antimony (Sb), which exhibit electronic configurations analogous to Pb and adopt the A_3_B_2_X_9_ structure [2], have emerged as promising alternatives. Both Bi and Sb possess a 6s^2^ lone pair, which is advantageous for achieving high photovoltaic performance [3]. These materials can crystallize in two distinct polymorphs: dimer (0D) and layered (2D) structures [4,5], with the specific form dictated by the size of the cation. Notably, Bi- and Sb-based perovskites exhibit superior air stability compared to their Sn- and Pb-based counterparts [6,7]. However, the 0D polymorph, characterized by a relatively large bandgap, is less suitable for solar cell applications [8,9].

Sb-based perovskites have garnered significant attention within the research community due to their relatively low excitonic binding energy compared to bismuth (Bi)-based counterparts. Pioneering work by Saparov et al. [4] demonstrated the replacement of Pb with Sb in PSCs, synthesizing Cs_3_Sb_2_I_9_ through a thermal evaporation process involving CsI and SbI_3_. Subsequent advancements involved substituting the A-site cation with organic groups, leading to the solution-processed synthesis of 0D-MA_3_Sb_2_I_9_ (MA = methylammonium), which achieved a modest power conversion efficiency (PCE) of approximately 0.5% [10]. Further exploration into Sb-based perovskites revealed the formation of layered phases using smaller cations such as Rb^+^ and NH_4_^+^, although the PCEs of these materials remained below 1%. Notably, the performance of MA_3_Sb_2_I_9_-based devices was significantly improved for both dimer (2.04% PCE with a short-circuit current density (*J*_sc_) of 5.14 mA/cm^2^) and layered phases (2.19% PCE with *J*_sc_ of 4.63 ± 0.41 mA/cm^2^) via solution processing. The enhanced photocurrent observed in devices incorporating organic cations (MA_3_Sb_2_I_9_) can be attributed to their broader light absorption range, particularly at longer wavelengths, compared to their inorganic counterparts, thereby improving overall light harvesting efficiency. However, the organic component, specifically MA, has been identified as a critical factor contributing to device instability, raising concerns regarding the long-term viability of such materials in PSCs [10,11].

It has been widely reported that formamidinium (FA) as a cation exhibits superior thermal stability compared to MA [12]. Consequently, significant efforts have been directed toward enhancing the stability and performance of PSCs by incorporating FA, either alone or in combination with MA, as well as through the mixing of halide anions. Notably, FA cations have been extensively utilized in high-performance Pb-based PSCs, where their larger ionic size facilitates a more optimal and red-shifted bandgap [12,13]. Building on this, the introduction of chloride (Cl) ions at the X-site in Sb-based perovskites has been proved to be effective in modulating crystal growth, improving surface morphology, and enhancing photoelectric properties, thereby promoting the formation of 2D perovskite structures. In this study, we developed FACl–Cs_3_Sb_2_I_9_ perovskite by introducing FACl onto the buried interface. This method effectively improved the film quality and passivated the interface defects of inorganic Cs_3_Sb_2_I_9_ PSCs. By constructing a device with the configuration FTO/TiO_2_/FACl–Cs_3_Sb_2_I_9_/Spiro-OMeTAD/Ag, we achieved a PCE of 1.66%. Furthermore, the device demonstrated remarkable stability under ambient conditions, attributed to the beneficial role of the FACl additive.

## 2. Materials and Methods

### 2.1. In This Experiment, All Reagents Were Used Directly Without Additional Purification

We employed cesium iodide (CsI, 99.99% Aladdin), antimony (III) iodide (SbI_3_, 99.98%, Aladdin), formamidinium chloride (FACl, 99.9%, Xi’an Yuri Solar Co., Ltd., Xi’an, China), 2,2′,7,7′-Tetrakis (*N*,*N*-dipmethoxyphenylamine)-9,9′-spirobifluorene (spiro-OMeTAD, 99.9%, Xi’an Yuri Solar Co., Ltd., Xi’an, China), *N*,*N*-dimethyl-formamide (DMF, J&K Scientific), isopropanol (IPA, Beijing Chemical Reagent Co., Ltd., Beijing, China) and hydrochloric acid (HCl, Beijing Chemical Reagent Co., Ltd., Beijing, China).

### 2.2. Preparation of the Electron Transport Layer

We added 30 μL hydrochloric acid (HCl) and 2 mL titanium tetraisopropyl alcohol to 30 mL isopropyl alcohol and stirred thoroughly. The FTO substrate was ultrasonically treated in detergent, deionized water, ethanol and isopropyl alcohol. After that, the substrate was then dried and cleaned using a nitrogen blower. The FTO glass was then treated with ozone for 15 min and placed on the homogenizer for spin-coating. The parameter of the spin-coating was set to 2000 rpm, the acceleration was 500 rpm, and the time was 60 s. The prepared titanium oxide solution was extracted by syringe and filtered through 0.45 μm polyether sulfone filter [11]. The filtered solution was placed on the FTO glass, and then the film was rotated. After that, the film was annealed on a hot table at 520 °C for 60 min.

### 2.3. FACl–Cs_3_Sb_2_I_9_ Synthesis

The Cs_3_Sb_2_I_9_ precursor solution was prepared based on methods described in the previous literature [14]. In short, it was prepared by dissolving 198 mg CsI (0.75 mmol) and 249 mg SbI_3_ (0.495 mmol) in 1 mL DMF solvent, and 30 µL HCl was added to obtain the layered phase by the solution method. A FACl–Cs_3_Sb_2_I_9_ layered phase film was obtained by spin-coating IPA (5 mg/mL) solution, dissolving a small amount of FACl on the buried interface.

### 2.4. Solar Cells Fabrication

Prior to device preparation, the FTO glass was treated with UV/O_3_ for 20 min. Next, for active layer deposition, the FTO-coated TiO_2_ sample was moved to an N_2_-filled glove box. Cs_3_Sb_2_I_9_ solution was then dropped onto the center of TiO_2_ substrates and spin-coated immediately at 4000 rpm for 30 s, with an acceleration rate of 1500 rpm/s. After 8 s of rotation, 150 μL isopropyl alcohol was dropped as an antisolvent. After spin-coating, the sample was annealed at 230 °C for 10 min to complete the fabrication of the perovskite layer. The hole transport layer was prepared by spiro-MeOTAD solution at 4000 rpm for 30 s (the spiro-MeOTAD preparation methods refer to previous work [14]). Finally, a ≈100 nm thick Ag top electrode was prepared via thermal evaporation.

### 2.5. Characterization

X-ray diffraction (XRD) with Cu Kα radiation (Bruker, D8 Discover, Karlsruher, Geman) was employed to investigate the crystal structure and phase confirmation. To study the optical absorption of Cs_3_Sb_2_I_9_ and FACl–Cs_3_Sb_2_I_9_ films, a UV–Visible (UV–Vis) spectrophotometer (PerkinElmer Instruments Lambda 950, Waltham, MA, USA) was applied. The Fourier transform infrared (FTIR) was obtained by making use of an FTIR spectrometer (FRITSCH invenio S, Grafenwald, Gemany). Scanning electron microscopy (SEM, SIGMA 500, Aachen, Gemany) was applied to characterize the morphology and microstructure of the films. The photoluminescence (PL) measurement was done using 405 nm pulsed lasers (Edinburgh instrument Ltd., FS5, Livingston, Kingdom of Scotland). Time resolved photoluminescence (TRPL, Edinburgh instrument Ltd., FS5, Livingston, Kingdom of Scotland) spectra were obtained using fluorescence spectra excited by a 340 nm laser. X-ray photoelectron spectroscopy (XPS) was performed with the Omicron ESCA Probe XPS spectrometer (Thermo Scientific ESCALAB 250Xi, Waltham, MA, USA). Ultraviolet photoelectron spectroscopy (UPS, He I excitation, 21.2 eV, referenced to the fermi edge of argon etched) was performed to calculate the energy levels. The current-voltage (*J-V*) curves were measured under a standard AM 1.5 G of 100 mW cm^−2^ solar simulator (Zolix (SOLAR IV-150A), Beijing, China). During efficiency measurements, the devices were covered with a metal mask with an aperture area of 0.1256 cm^2^. The incident photon-to-electron conversion efficiency (IPCE) was obtained using a solar cell measurement system. Measurements of transient photovoltage (TPV) and transient photocurrent (TPC) were taken through a transient photovoltage test system. TPC was determined under short-circuit conditions, while TPV was established under open-circuit conditions.

## 3. Results and Discussion

### 3.1. Structure Formation and Layered Phase Deposition

To achieve a layered phase through a solution-based process, we previously developed a straightforward and efficient methodology. Initially, we demonstrated that incorporating HCl into the precursor solution could effectively alter the structural properties of Cs_3_Sb_2_I_9_ films [14]. As illustrated in Figure S1, the diffraction peak positions of the film without adding HCl were 12.7°, 24.0°, and 25.2°—a typical 0D structure. However, no matter how much HCl was added, the position of the diffraction peak of the thin films changed to 25.7°and 29.2°—characteristic diffraction peaks of 2D structures. Furthermore, we observed that increasing the HCl concentration led to a significant narrowing of the full width at half maximum (FWHM) of the XRD peak, indicating a notable enhancement in the crystallinity of the films. This finding underscores the critical role of HCl in optimizing the structural and crystalline quality of Cs_3_Sb_2_I_9_ perovskite films.

**Figure 1:**
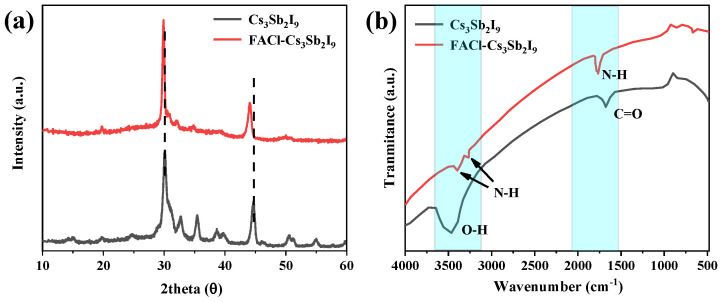
(**a**) XRD patterns of Cs_3_Sb_2_I_9_ and FACl–Cs_3_Sb_2_I_9_ (**b**) FTIR spectra of pristine and FACl-treated sample. The peaks at 1764, 3276, and 3402 cm^−1^ indicate N-H bonds.

In this study, we introduced a small amount of FACl, dissolved in IPA, to achieve a uniform and compact perovskite film. (For detailed experimental procedures, refer to the experimental section.) Prior to assembling the complete photovoltaic device, the structural and morphological properties of both FACl-treated and pristine Cs_3_Sb_2_I_9_ films were thoroughly investigated [13,15]. The crystal structures of the films, before and after FACl treatment, were analyzed using XRD, as depicted in Figure 1a. Both films exhibited a layered phase; however, the peak intensity ratio of the FACl-treated film was significantly higher than that of the pristine film. The results showed that the grain growth of the FACl-treated sample was accelerated. Additionally, the XRD peaks of the FACl-treated films exhibited a shift toward lower angles, indicating an increase in grain size. Furthermore, the FWHM of the XRD peaks decreased for the FACl-treated films, confirming enhanced crystallinity [12,16,17].

**Figure 2:**
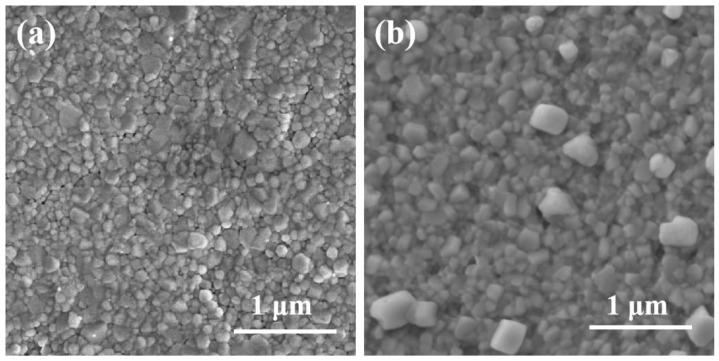
FACl-incorporated images of pristine Cs_3_Sb_2_I_9_ (**a**) and FACl–Cs_3_Sb_2_I_9_ film (**b**).

To further characterize the films, an FTIR test was employed. As shown in Figure 1b, the FTIR spectra confirmed the presence of FA in the FACl–Cs_3_Sb_2_I_9_ film, with distinct peaks observed at 1764 cm^−1^ and 3276 cm^−1^, corresponding to N-H vibrations. The stretching vibration bond at 1679 cm^−1^ was attributed to C=O, while the broad peak at 3463 cm^−1^ in the pristine Cs_3_Sb_2_I_9_ spectra was assigned to O-H. Notably, these peaks (C=O and O-H) disappeared in the FACl-incorporated sample, indicating the successful modification of the film. To verify the incorporation of Cl ions, XPS was conducted. As illustrated in Figure S2, a distinct Cl 2p signal was detected in the FACl-treated film, confirming the presence of Cl within the Cs_3_Sb_2_I_9_ lattice [18,19]. Moreover, SEM was utilized to analyze the surface morphology of the films. As shown in Figure 2, both pristine and FACl-treated Cs_3_Sb_2_I_9_ films exhibited high surface coverage. However, the FACl-treated film displayed larger grain sizes compared to the pristine film, consistent with previous reports that Cl incorporation promoted grain growth in perovskite films [14,20,21]. This observation further corroborated the successful integration of Cl ions into the Cs_3_Sb_2_I_9_ structure [22].

**Figure 3:**
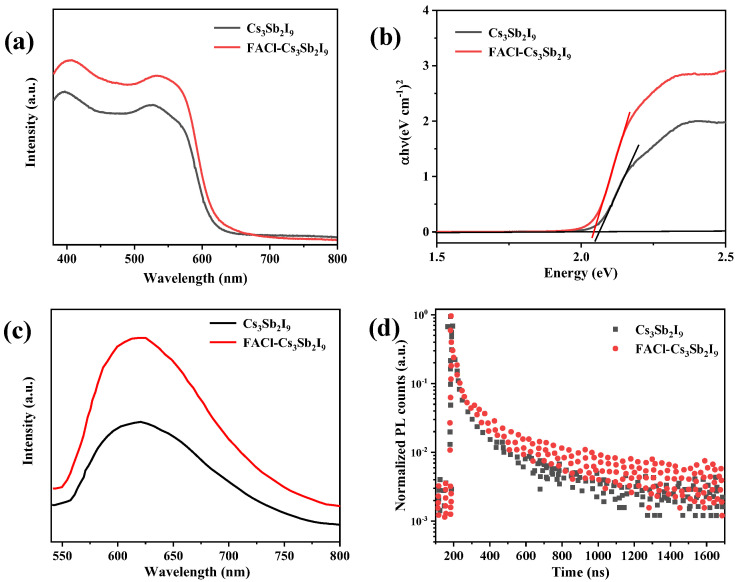
(**a**) UV–Vis absorption spectra of Cs_3_Sb_2_I_9_ and FACl–Cs_3_Sb_2_I_9_. (**b**) Band gap of pristine and FACl–Cs_3_Sb_2_I_9_. (**c**) PL of spectra. (**d**) Time-resolved PL (trPL) pristine Cs_3_Sb_2_I_9_ (black) and FACl–Cs_3_Sb_2_I_9_ (red).

The optical properties of both pristine and FACl-incorporated films were systematically investigated using UV–Vis spectroscopy and PL measurements. The FACl-incorporated film demonstrated a significantly enhanced absorption intensity while maintaining a slightly decreasing absorption edge, consistent with the bandgap values derived from the Tauc plot (Figure 3a,b). In the FACl-incorporated film, the PL emission peak of intensity was enhanced, along with a slight redshift in the emission peak location, as shown in Figure 3c. These data indicated that the FACl–Cs_3_Sb_2_I_9_ film had a higher absorption strength, which was conducive to the generation of higher photocurrent [18,23,24]. To gain deeper insights into the film quality and exciton dynamics, TRPL measurements were conducted. Figure 3d presented the TRPL decay curves for both pristine and FACl-treated films. Analysis of the decay profiles revealed exciton lifetimes of 7.1 ns for the pristine Cs_3_Sb_2_I_9_ film and 8.7 ns for the FACl–Cs_3_Sb_2_I_9_ film. The longer exciton lifetime observed in the FACl-treated film indicated superior film quality with fewer defect states. The beneficial role of FACl in enhancing the photoelectric properties of the perovskite layer was further confirmed [12,25,26].

**Figure 4:**
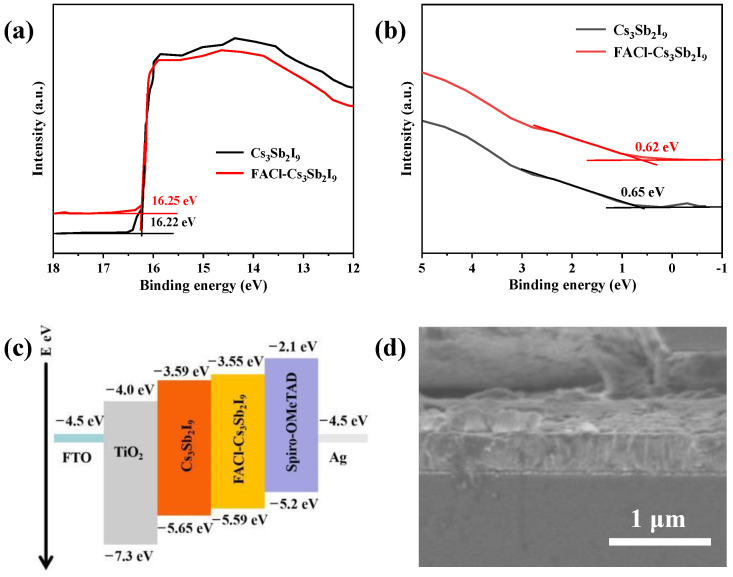
(**a**,**b**) UPS expanded spectra of both Cs_3_Sb_2_I_9_ and FACl–Cs_3_Sb_2_I_9_ samples. (**c**) The energy-level diagram. The scale bar shows each parameter’s conduction and valance band. (**d**) Cross-section SEM image shows the compactness of FACl–Cs_3_Sb_2_I_9_.

To investigate the impact of FACl incorporation on the energy levels of Cs_3_Sb_2_I_9_, a UPS test was employed. The UPS spectra of both pristine and FACl-incorporated Cs_3_Sb_2_I_9_ films are presented in Figure 4a,b. For the pristine Cs_3_Sb_2_I_9_ film, the high and low binding energy cutoffs were observed at 16.22 eV and 0.65 eV, respectively. Following FACl treatment, these values shifted to 16.25 eV and 0.62 eV, respectively. The conduction band (CB) positions were determined by adding the bandgap values to the valence band (VB) energies. The calculated CB values for the pristine and FACl-incorporated films were 3.59 eV and 3.55 eV, respectively. All corresponding energy-level values are summarized in Figure 4c. The reduced energy gap between the VB and the Fermi level in the FACl–Cs_3_Sb_2_I_9_-based device was anticipated to enhance carrier concentration and hole mobility. Additionally, the upward shift in the energy levels of the FACl–Cs_3_Sb_2_I_9_ film reduces the energy-level mismatch between the absorption layer and the hole transport layer, which is expected to contribute to an increased open-circuit voltage (*V*_oc_) in the photovoltaic device [14,27,28]. Figure 4d displays a cross-sectional image of the fabricated device, revealing a total thickness of approximately 450 nm.

### 3.2. The Performance of Device and Physical Characterization

**Figure 5:**
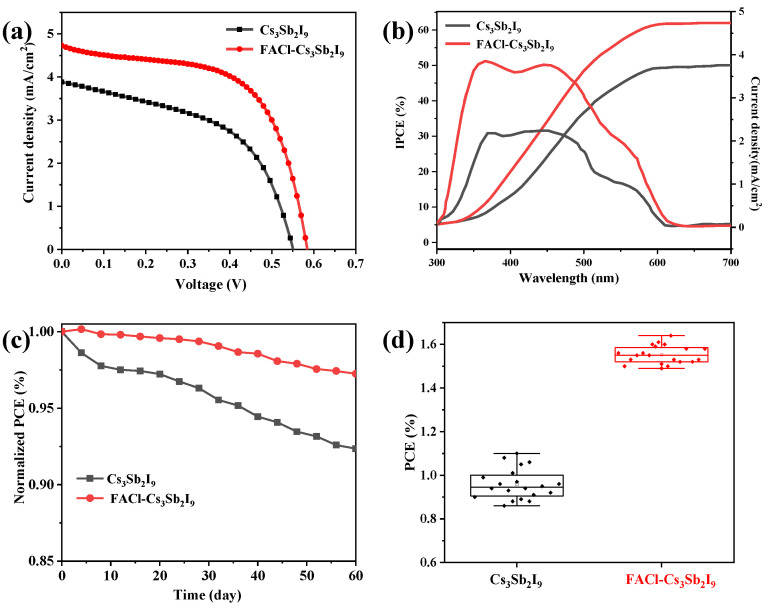
(**a**) The optimal *J*-*V* curves of PSCs based on Cs_3_Sb_2_I_9_ and FACl–Cs_3_Sb_2_I_9_ film. (**b**) IPCE spectra of Cs_3_Sb_2_I_9_ pristine and FACl-incorporated sample and the integrated *J*_sc_. (**c**) Environmental stability of the pristine and FACl–Cs_3_Sb_2_I_9-_incorporated devices. (**d**) PCE box statistics of 20 Cs_3_Sb_2_I_9_ devices and FACl–Cs_3_Sb_2_I_9_ devices.

A simple planar device architecture, FTO/TiO_2_/perovskite/Spiro-OMeTAD/Ag, was employed to fabricate photovoltaic devices. The performance of solar cells based on pristine Cs_3_Sb_2_I_9_ and FACl-treated Cs_3_Sb_2_I_9_ films was evaluated. Figure 5a illustrates the *J*-*V* characteristics of the champion devices under AM 1.5G illumination (100 mW/cm^2^). The FACl-incorporated champion device achieved a PCE of 1.66%, with an *V*_oc_ of 0.58 V, a short-circuit current density (*J*_sc_) of 4.73 mA/cm^2^, and a fill factor (FF) of 60.5%. In contrast, the pristine device exhibited a PCE of 1.10%, with a *V*_oc_ of 0.55 V, a *J*_sc_ of 3.89 mA/cm^2^, and an FF of 51.4%. In order to directly compared the performance of our prepared devices, the performance of some Sb-based solar cells in recent years was listed in Table 1. Additionally, the forward and reverse scan *J*-*V* curves, shown in Figure S3, revealed minimal hysteresis in the Sb-based solar cells. Due to the mismatch of energy levels between the Sb-based perovskite film and the charge transport layer, charge carrier accumulation occurs at the interface during charge transport. This may be one of the main reasons for the hysteresis of the Sb-based perovskite device. In the future, if the charge transport layer is more suitable for Sb-based perovskite films, it is expected to further reduce or even eliminate the hysteresis. The photovoltaic parameters of the devices are summarized in Table S1. The slight improvements in FF and *V*_oc_ can be attributed to the suppression of non-radiative recombination and interfacial defects due to FACl incorporation [29,30,31], which is consistent with the findings from PL and TRPL measurements.

The increase in *J*_sc_ from 3.89 to 4.73 mA/cm^2^ was further corroborated by the IPCE spectra of the pristine and FACl–Cs_3_Sb_2_I_9_ devices, as depicted in Figure 5b. The IPCE spectra demonstrated that the enhanced photocurrent arises from improved light harvesting across the broadband spectrum, which was a consequence of the superior film quality and reduced bandgap achieved through FACl treatment [14,32]. The results from the solar simulator were in excellent agreement with the IPCE measurements. Furthermore, the UV–Visible absorption spectra (Figure 3a) corroborated this trend, showing enhanced absorption across the entire range, which confirmed that the improvement in *J*_sc_ was primarily due to increased light absorption [33,34,35]. This enhancement in absorption strength is attributed to the larger grain size and improved crystallinity resulting from FACl treatment. Since the radius of FA ions is larger than that of Cs ions, the introduction of FA ions into the lattice of perovskite will increase the light absorption range of the film, which has been widely used in lead-based perovskite [17]. The introduction of FA ions into Cs_3_Sb_2_I_9_ films can also increase the light absorption range of the film, thereby increasing *J*_sc_.

In addition to its excellent photovoltaic performance, the incorporation of FACl significantly enhances the long-term stability of Cs_3_Sb_2_I_9_-based PSCs. Both Cs_3_Sb_2_I_9_ and FACl–Cs_3_Sb_2_I_9_ devices, without any additional treatment, were stored in a nitrogen (N_2_) glovebox environment and subsequently tested for operational stability under ambient conditions [36]. As illustrated in Figure 5c, the FACl–Cs_3_Sb_2_I_9_ device demonstrated superior stability compared to its pristine counterpart. After two months of storage, the FACl–Cs_3_Sb_2_I_9_ device exhibited less than 3% degradation in PCE, whereas the pristine Cs_3_Sb_2_I_9_ device experienced more than 5% degradation. To demonstrate that FACl processing can improve stability under different conditions, we encapsulated the device and tested it. Figure S4 shows the stability of the encapsulated device. Both the Cs_3_Sb_2_I_9_ device and FACl–Cs_3_Sb_2_I_9_ device showed good stability. After 50 days of storage, the performance of the Cs_3_Sb_2_I_9_ device remained at 97%, while the performance of the FACl–Cs_3_Sb_2_I_9_ device remained at 98%. Therefore, this method can enhance the stability of different forms of devices. Furthermore, Figure 5d highlights the reproducibility of the Cs_3_Sb_2_I_9_ and FACl–Cs_3_Sb_2_I_9_ devices. The statistical distribution of PCE for the FACl–Cs_3_Sb_2_I_9_ device was narrower and more consistent compared to the pristine Cs_3_Sb_2_I_9_ device, indicating better reproducibility. These results underscore the dual benefits of FACl incorporation: enhanced stability and improved device reproducibility. This is expected to be a promising strategy for advancing lead-free PSCs [37,38,39].

**Table 1:** Summary of performance of Sb-based PSCs.

	*J*_sc_ (mA/cm^2^)	*V*_oc_ (V)	FF (%)	η (%)	Ref.
Cs_3_Sb_2_I_9_	0.13	0.40	58.0	0.03	[39]
MA_3_Sb_2_I_9_	1.0	0.89	55	0.49	[10]
MA_3_Sb_2_I_9_	1.48	0.74	52	0.57	[23]
Cs_3_Sb_2_I_9_	2.34	0.62	46.18	0.67	[2]
Cs_3_Sb_2_I_9_	3.55	0.61	55.8	1.21	[13]
Cs_3_Sb_2_I_9_	5.31	0.72	38.97	1.49	[8]
FAI-Cs_3_Sb_2_I_9_	5.57	0.62	51.4	1.76	[12]
Cs_3_Sb_2_I_9_	5.40	0.80	54.9	2.48	[14]
MA_3_Sb_2_I_9_	6.64	0.70	59.6	2.77	[29]
FACl–Cs_3_Sb_2_I_9_	4.73	0.58	60.5	1.66	This work

**Figure 6:**
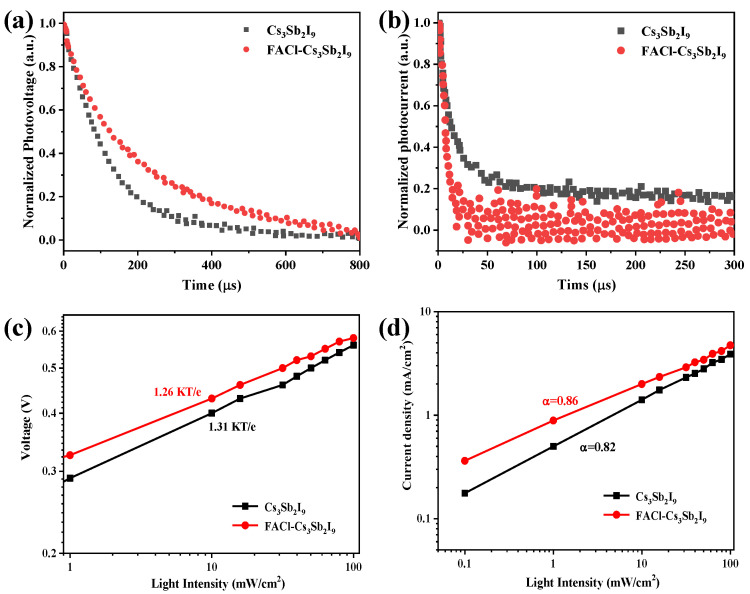
(**a**) Normalized TPV of pristine and FACl-incorporated sample. (**b**) Normalized TPC decay of pristine (red) and FACl–Cs_3_Sb_2_I_9_ devices. Light intensity dependence of *V*_oc_ (**c**) and *J*_sc_ (**d**) for the Cs_3_Sb_2_I_9_ and FACl–Cs_3_Sb_2_I_9_ device.

To elucidate the underlying reasons for the enhanced performance of the representative device, TPV and TPC measurements were taken. As shown in Figure 6a, TPV measurements taken under open-circuit conditions showed that the recombination lifetime (τ_r_) of the original Cs_3_Sb_2_I_9_ device was 145 µs, which was significantly shorter than that of the FACl–Cs_3_Sb_2_I_9_ device (257 µs). This trend aligned with the findings from the TRPL measurements [12,18,40]. The TPV results suggested that the FACl–Cs_3_Sb_2_I_9_ device had a lower defect concentration compared to the pristine Cs_3_Sb_2_I_9_ device. Similarly, TPC measurements taken under short-circuit conditions demonstrated that the FACl–Cs_3_Sb_2_I_9_-based device exhibited a faster charge transfer time (8.7 µs) than the pristine Cs_3_Sb_2_I_9_ device (9.8 µs), as depicted in Figure 6b. The improvement in charge transfer kinetics can be attributed to the optimized band alignment between the valence band of the active absorber layer and the charge transport layer [20,41], as confirmed by the UPS analysis above.

The recombination of carrier at the buried interface of PSCs was also studied. Figure 6c shows the variable-light-intensity *J*-*V* measurements of the Cs_3_Sb_2_I_9_ and FACl–Cs_3_Sb_2_I_9_ PSCs. A slope deviation of 1 KT/e indicates the possibility of trap-assisted recombination under open-circuit conditions. The slope of the Cs_3_Sb_2_I_9_ device is 1.31 KT/e, where e is the elementary charge, K is the Boltzmann constant, and T is the Kelvin temperature. However, the slope value of the FACl–Cs_3_Sb_2_I_9_ device is 1.26 KT/e. When the slope is reduced, the FACl–Cs_3_Sb_2_I_9_ device can effectively suppress the trap-assisted recombination at the buried interface. Reducing recombination is beneficial to the improvement of *V*_oc_. At the same time, it may also explain why the hysteresis based on FACl–Cs_3_Sb_2_I_9_ PSCs is smaller [42]. *J*_sc_ and I^α^ under short-circuit conditions are shown in Figure 6d, where the data are expressed on a log–log scale and complied with the power law. For Cs_3_Sb_2_I_9_ and FACl–Cs_3_Sb_2_I_9_ devices, the index values are 0.82 and 0.86, respectively. The value of FACl–Cs_3_Sb_2_I_9_ PSC is larger, indicating that the device has less bimolecular recombination and more carriers can be transported to the charge transport layer before recombination. Modifying FACl at the buried interface can reduce trap-assisted recombination and the bimolecular recombination of devices, thus boosting charge transport in solar cells.

These results collectively highlighted the beneficial effects of FACl incorporation in reducing defect states and enhancing charge transport, thereby contributing to the overall improvement in device performance [14,18,43]. To be specific, when FACl is used to modify the buried interface of the device, FACl will be doped onto the Sb-based film. Since FA ions are larger than Cs ions, the film-doping FA can improve the light absorption capacity. The addition of Cl ions in the film can increase the grain size and reduce the film defects. Therefore, the *J*_sc_ of the device can be improved by using FACl to treat the buried interface. In addition, the treatment can also adjust the level position of the perovskite film, reduce the level mismatch between the Sb-based perovskite layer and the charge transport layer, optimize the interface charge transport, and finally increase the *V*_oc_ of the device.

## 4. Conclusions

In this study, we demonstrated that modifying the buried interface with FACl can significantly enhance the performance of Sb-based PSCs without compromising their stability. Specifically, the modification of the buried interface with FACl optimizes film morphology and enhances the transport of photo-generated carriers. At the same time, this treatment can also reduce the energy-level mismatch and interface recombination between the perovskite layer and the charge transport layer. Therefore, this method can improve the overall performance of the device. The FACl–Cs_3_Sb_2_I_9_ device achieved a PCE of 1.66%, which was 50% higher than that of the pristine Cs_3_Sb_2_I_9_ device. The layered phase of Cs_3_Sb_2_I_9_ perovskite holds significant potential for applications in other optoelectronic devices in the future. This study provides an effective approach for further enhancing the performance of Sb-based perovskite optoelectronic devices.

## Data Availability

The original contributions presented in the study are included in the article, further inquiries can be directed to the corresponding author.

## Supplementary Materials

The following supporting information can be downloaded at: https://www.mdpi.com/article/10.3390/mi16040379/s1. Figure S1. XRD patterns of the films deposited from different concentration of HCl additive. The arrows mark the characteristic diffraction peaks of the layered phase and the dimer phase. The layered phase Cs_3_Sb_2_I_9_ was successfully prepared by adding HCl. Figure S2. The core level XPS spectra of Cl 2p of both the Cs_3_Sb_2_I_9_ film and FACl-Cs_3_Sb_2_I_9_ film. In the FACl-Cs_3_Sb_2_I_9_ film, the Cl signal can be clearly characterized, indicating that the Cl element incorporated into the Sb-based film. Figure S3. Forward and reverse scans of *J*-*V* curves of PSCs devices based on the Cs_3_Sb_2_I_9_ film and FACl-Cs_3_Sb_2_I_9_ film. Figure S4. Stability of Cs_3_Sb_2_I_9_ and FACl-Cs_3_Sb_2_I_9_ encapsulated devices. Table S1. Summary of photovoltaic parameters of PSCs based on the Cs_3_Sb_2_I_9_ film and FACl-Cs_3_Sb_2_I_9_ film.
