# Supplementary material for: FACl as a Bifunctional Additive to Enhance the Performance of Lead-Free Antimony-Based Perovskite Solar Cells
Source: Micromachines (Basel). 2025 Mar 27;16(4):379. doi: 10.3390/mi16040379 (PMC12029204; doi:10.3390/mi16040379)
Supplement: Supplementary file 1 [file micromachines-16-00379-s001.zip › micromachines-3514364-supplementary.pdf]

FACl as a Bifunctional Additive to Enhance the Performance of Lead-Free Antimony-Based Perovskite Solar Cells

Xinyu Gao [1], Zihao Gao [1], Zhen Sun [1], Ping Song [1], Xiyuan Feng [2,*] and Zhixin Jin [1,*]

[1] School of Science, Yanshan University, Qinhuangdao 066004, China
[2] School of Microelectronics, Northwestern Polytechnical University, Xi'an 710129, China
* Correspondence: fengxy@nwpu.edu.cn (X.F.); jinzx@ysu.edu.cn (Z.J.)

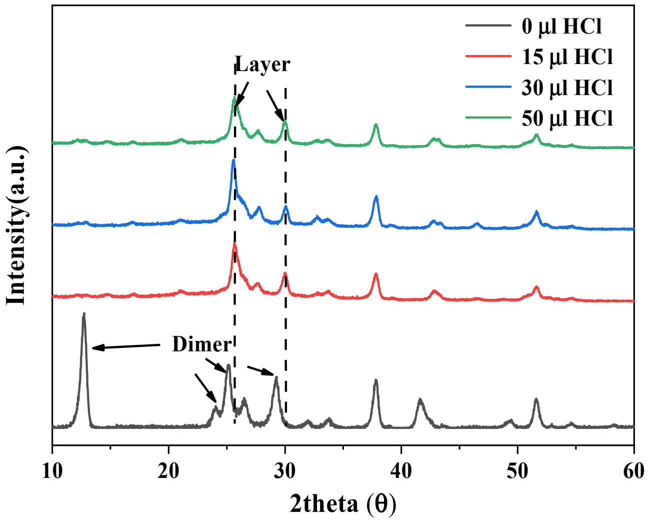

Figure S1. XRD patterns of the films deposited from different concentration of HCl additive. The arrows mark the characteristic diffraction peaks of the layered phase and the dimer phase. The layered phase $Cs_3Sb_2I_9$ was successfully prepared by adding HCl.

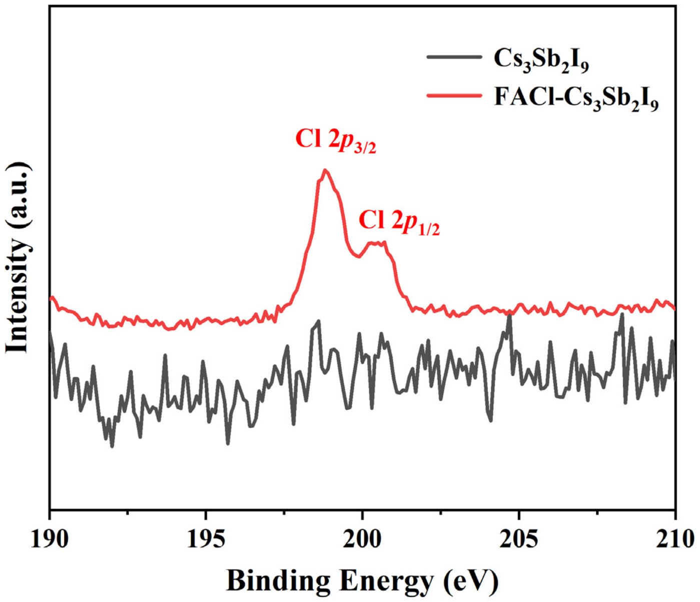

Figure S2. The core level XPS spectra of Cl 2p of both the $Cs_3Sb_2I_9$ film and FACl-$Cs_3Sb_2I_9$ film. In the FACl-$Cs_3Sb_2I_9$ film, the Cl signal can be clearly characterized, indicating that the Cl element incorporated into the Sb-based film.

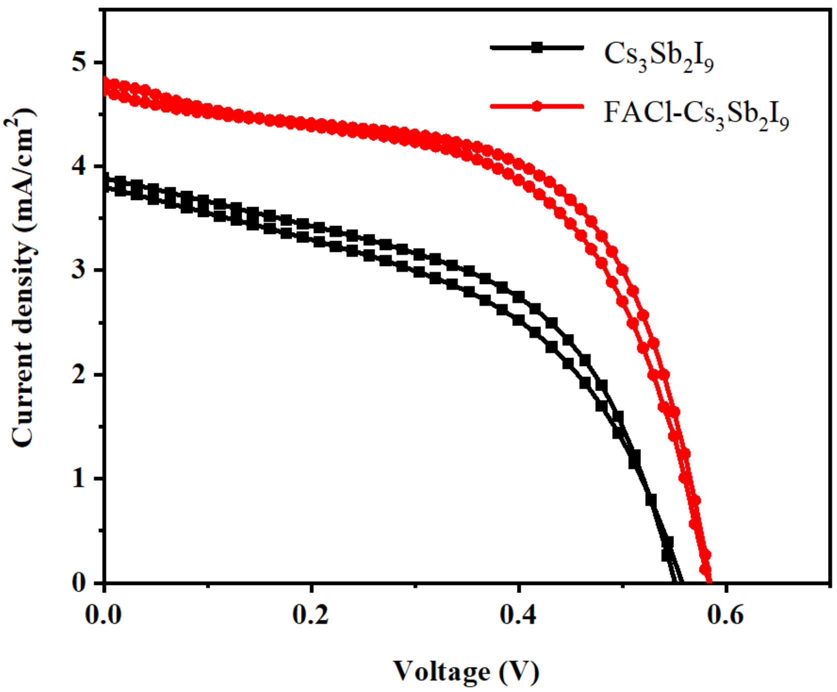

Figure S3. Forward and reverse scans of *J*–*V* curves of PSCs devices based on the $Cs_3Sb_2I_9$ film and FACl-$Cs_3Sb_2I_9$ film.

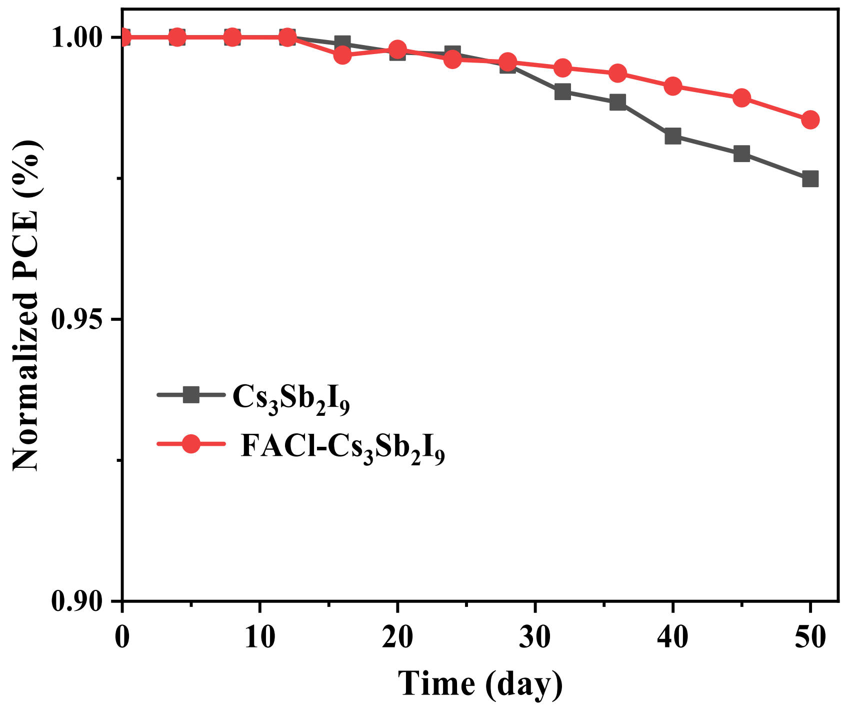

Figure S4. Stability of $Cs_3Sb_2I_9$ and FACl-$Cs_3Sb_2I_9$ encapsulated devices.

Table S1. Summary of photovoltaic parameters of PSCs based on the $Cs_3Sb_2I_9$ film and FACl-$Cs_3Sb_2I_9$ film.

	J_{sc} (mA/cm^2)	V_{oc} (V)	FF (%)	η (%)
$Cs_3Sb_2I_9$	3.89/3.80	0.55/0.56	51.4/51.7	1.10/1.01
FACl-$Cs_3Sb_2I_9$	4.73/4.81	0.58/0.58	60.5/56.2	1.66/1.57
